# Detection of *Cereibacter azotoformans*-YS02 as a Novel Source of Coenzyme Q10 and Its Metabolic Analysis

**DOI:** 10.3390/antiox14040429

**Published:** 2025-04-01

**Authors:** Meijie Song, Qianqian Xu, Rifat Nowshin Raka, Chunhua Yin, Xiaolu Liu, Hai Yan

**Affiliations:** 1 School of Chemistry and Biological Engineering, University of Science and Technology Beijing, Beijing 100083, China; meijie_song@126.com (M.S.); qianqianxu@ustb.edu.cn (Q.X.); chyin@ustb.edu.cn (C.Y.); xiaoluliu@ustb.edu.cn (X.L.); 2 Institute of Apicultural Research, Chinese Academy of Agricultural Sciences, Beijing 100093, China; rnraka92@gmail.com

**Keywords:** *Cereibacter azotoformans*-YS02, genomic analysis, CoQ10 biosynthesis, UPLC-Q-Exactive Orbitrap MS, metabolomics analysis

## Abstract

Coenzyme Q10 (CoQ10), a high-value-added nutraceutical antioxidant, exhibits an excellent ability to prevent cardiovascular disease. Here, a novel *Cereibacter azotoformans* strain, designated YS02, was isolated for its ability to produce CoQ10 and genetically characterized by whole genome sequencing (WGS). The CoQ10 biosynthesis and metabolism differences of YS02 under various culture conditions were also systematically investigated. Phylogenetic analysis based on 16 S rRNA genes, along with taxonomic verification using average nucleotide identity (ANI) analysis, confirmed its classification as *C. azotoformans*. Enzymatic genes *dxs*, *dxr*, *idi, ubiA*, and *ubiG* were annotated in YS02, which are critical genetic hallmarks for CoQ10 biosynthesis. Under aerobic–dark cultivation, YS02 grows well, and CoQ10 production can reach 201 mg/kg. A total of 542 small-molecule metabolites were identified from YS02 in aerobic–dark and anaerobic–light cultivation via ultra-high performance liquid chromatography–coupled quadrupole orbitrap high-resolution mass spectrometry (UPLC-Q-Exactive Orbitrap MS). Additionally, 40 differential metabolites were screened through multivariate statistical analysis. Metabolic pathway analysis revealed that the biosynthesis of phenylalanine, tyrosine, and tryptophan might be latent factors influencing CoQ10 production discrepancies within YS02 under both cultural modes. These findings represent new insights into the metabolic mechanism of YS02 and underscore its potential as an alternative strain source for industrial CoQ10 production, enriching the existing resources.

## 1. Introduction

*Cereibacter azotoformans* is a purple non-sulfur bacteria (PNSB) first reported in 1995 [1]. As a type of facultative anaerobic gram-negative bacterium, *C. azotoformans* has the capability to live in diverse environmental conditions. It can grow aerobically as a chemoheterotroph and anaerobically in light as a photoheterotroph [2,3]. According to previous studies, *C. azotoformans* exhibits a complex genome structure and substantial metabolic multifunction [4,5,6]. It has been extensively utilized in agricultural income enhancement, aquaculture, ecological restoration, and other fields [6,7,8,9]. Moreover, the extract of *C. azotoformans* was mentioned as a health supplement for humans and animals in Japan, aimed at modulating immune responses in vivo [10]. For bioeconomic purposes, this bacterium has continuously been subject to attempts at synthesizing vehicles for relevant compounds like carotenoids [11], novel lipo-oligosaccharide [12], and hydrogen [2].

CoQ10 is a Food and Drug Administration (FDA)-approved dietary supplement with an estimated 10% annual growth rate in the global market [13]. As a lipid-soluble molecule, CoQ10 possesses three states: the reduced form (ubiquinol), the semiquinone radical form, and the oxidized form (ubiquinone) [14]. CoQ10 has a physiological key effect on mitochondrial bioenergetics and demonstrates strong antioxidant activity [15]. It participates in the electron transport chain, boosts ATP production, scavenges free radicals, and protects cell membranes and proteins from oxidative damage [16]. These prominent properties of CoQ10 sparked the high consumption demand. Whereas CoQ10 naturally occurs in some foods, the daily dietary intake is still insufficient to compensate for the age-related level reduction. So, the exogenous supply of CoQ10 from replenishment sources became necessary. A multitude of research has linked CoQ10 deficiencies to a variety of diseases, including cardiovascular diseases, neurodegenerative disorders, metabolic syndrome, diabetes, and cancer [17,18,19]. It is currently the third most consumed nutritional supplement and is regarded as a potential candidate for the treatment of different diseases and health conditions [20].

The production of CoQ10 generally consists of chemical synthesis, microbial biosynthesis, and extraction from biological tissues [16,21]. By far, microbiological fermentation technology has been deemed as a predominant approach for improving CoQ10 production. In particular, CoQ10 synthesized by microbial fermentation is an all-trans conformation without producing other optical isomers and is called reduced CoQ10 which is more easily absorbed by the human body [22,23]. Consequently, it is reasonable to investigate and develop natural strains that produce CoQ10. At present, *Rhodobacter sphaeroides* is the most productive native host and is a good option for industrial-scale production of CoQ10 [20,21]. Some previous studies have endeavored to adopt the strategy based on metabolic control of the fermentation to enhance the yield of CoQ10 in *R. sphaeroides*. For example, [24] compares the results of aerobic–dark and anaerobic–light cultivation with different quantities of dissolved oxygen. Another strategy is to use glucose-fed batch fermentation in combination with phosphate limitation (lower phosphate or without the addition of inorganic phosphate) [25] or to analyze the impact of different pH regulators (NH_3_·H_2_O and CaCO_3_) on the metabolic flux of CoQ10 [23]. *R. sphaeroides* is the closest neighbor of *C. azotoformans* in genetic relationships/associations [26]. Nevertheless, there is no report focusing on the production of CoQ10 using *C. azotoformans* and a lack of evidence of the metabolic process of CoQ10 under different culture modes.

The genome conveys crucial information about microbial physiology and biochemistry, essential for understanding fermentation biology. Untargeted metabolomics can measure metabolites, capturing unique compound profiles and secondary metabolite variabilities due to genome, environmental, and food processing factors [27]. In the present work, the biosynthesis gene of CoQ10 in YS02 and its molecular mechanism were annotated by whole genome sequencing (WGS). Furthermore, the concentration of CoQ10 in YS02 was determined by a developed high-performance liquid chromatography (HPLC) analysis method. We collected YS02 samples under four diverse culture modes and monitored the growth and the CoQ10 content of YS02 cells at various culture times to identify the competitive production method and provide guidance for follow-up applications. A sensitive and high-efficiency analysis method based on ultra-high performance liquid chromatography–coupled quadrupole orbitrap high-resolution mass spectrography (UPLC-Q-Exactive Orbitrap MS) was developed to further reveal the metabolic differences between whole growth phases of aerobic–dark and anaerobic–light cultivation of YS02. This approach can identify a wide range of small molecules, including bioactive secondary metabolites, providing valuable insights into the metabolic pathways involved in CoQ10 biosynthesis. The objective of the metabolomics analysis was to identify the advantageous metabolic pathway to pinpoint potential targets for enhancing CoQ10 production. This study systematically demonstrated the molecular and chemical basis of *C. azotoformans*-YS02 to generate CoQ10 as a novel strain and thereby laid the groundwork for developing better bioprocess methods and metabolically engineered strains to reach the commercial level of production.

## 2. Materials and Methods

### 2.1. Materials, Reagents, and Culture Media

CoQ10 (≥98%) was obtained from Shanghai Aladdin Biochemical Technology Co., Ltd. (Shanghai, China). HPLC-grade methanol, HPLC-grade ethanol, and HPLC-grade acetonitrile were purchased from Fisher Chemicals (Fairlawn, NJ, USA). Ultra-pure water was prepared using a Milli-Q water system (Milli-pore–Waters, Milford, MA, USA). Formic acid (MS-grade) was obtained from Thermo Fisher Scientific (Shanghai, China). The anaeropack (Oxoid^TM^ AnaeroGen^TM^ 2.5 L) was purchased from Beijing BioDee Biotechnology Co., Ltd. (Beijing, China).

A strain of facultative photoautotroph was isolated from river channel silt (Beijing, China). We identified this novel strain as *C. azotoformans*-YS02 (Figure S1) and preserved it in the China General Microbiological Culture Collection Center (CGMCC) under the number 31994. The complex culture media contained glucose (10 g/L), brown sugar (20 g/L), yeast extract powder (10 g/L), peptone (15 g/L), glycerol (2 g/L), ammonium citrate tribasic (2 g/L), Na_2_CO_3_ (1.2 g/L), KH_2_PO_4_ (1.0 g/L), MgSO_4_ (0.5 g/L), and multivitamin B tablet (0.1 piece/L). All components were dissolved in deionized water. The solid medium was formulated by incorporating 1.8% agar powder into the liquid medium. Both the medium and the culture vessels were sterilized in an autoclave at 115 °C for 30 min. After sterilization, the medium was cooled to room temperature and inoculated with YS02 cells (1%) from the logarithmic growth phase. The initial cell density was approximately 1.5 to 2 × 10^6^ CFU/mL, as determined by the plate count method. The inoculated cultures were then incubated in an orbital shaker at 30 °C and 200 rpm. The corresponding anaerobic–light cultivation was operated at the illumination incubator. The sample surface was illuminated using an incandescent lamp positioned at a distance of 10 cm, and the illumination was in the range of 1.2~1.5 kilolux (klx).

A two-stage culture process was implemented. The switch time occurred during the exponential phase, about 24 h post-inoculation. The four diverse culture modes were anaerobic–light, 24 h of anaerobic–light followed by 48 h of aerobic–dark, aerobic–dark, and 24 h of aerobic–dark followed by 48 h of anaerobic–light.

### 2.2. WGS Analysis of C. azotoformans-YS02

Firstly, the genomic DNA of *C. azotoformans*-YS02 was isolated using the Wizard^®^ Genomic DNA Purification Kit (Promega, Madison, WI, USA) as per the manufacturer’s instructions. The purified DNA was then quantified using a TBS-380 fluorometer (Turner BioSystems Inc., Sunnyvale, CA, USA). Only high-quality DNA samples, characterized by an OD260/280 ratio of 1.8 to 2.0 and a quantity exceeding 20 µg, were selected for subsequent analyses.

Secondly, genome sequencing was performed using both the PacBio RS II Single Molecule Real-Time (SMRT) (Menlo Park, CA, USA) and Illumina platforms (San Diego, CA, USA). Illumina sequencing was employed to assess the genome’s complexity. For library construction, at least 1 µg of genomic DNA was fragmented into 400–500 bp pieces using a Covaris M220 Focused Acoustic Shearer (Waltham, MA, USA). The sequencing libraries were then prepared using the NEXTflex™ Rapid DNA-Seq Kit (Bioo Scientific, Austin, TX, USA). Specifically, the 5′ ends of the fragments were end-repaired and phosphorylated, followed by A-tailing of the 3′ ends and ligation of sequencing adapters. The adapter-ligated products were enriched via PCR. These libraries were subsequently used for paired-end sequencing (2 × 150 bp) on an Illumina NovaSeq X Plus platform. For PacBio sequencing, 15 µg of DNA was sheared using a Covaris g-TUBE (Waltham, MA, USA) at 6000 rpm for 60 s in an Eppendorf 5424 centrifuge (Hamburg, Germany). The resulting fragments were purified, end-repaired, and ligated to SMRTbell sequencing adapters (Menlo Park, CA, USA) following the manufacturer’s protocol. The sequencing library was purified three times using Agencourt AMPure XP beads (Beckman Coulter, Pasadena, CA, USA) at 0.45× volume. Next, a ~10 kb insert library was prepared and sequenced on a single SMRT cell using standard procedures.

Lastly, the data generated from PacBio and Illumina platform were used for bioinformatics analysis. Genome assembly, gene prediction, and annotation were performed using the I-Sanger Cloud Platform (https://cloud.majorbio.com) (accessed on 31 May 2024) from Shanghai Majorbio (Shanghai, China).

The 16S rRNA gene sequence (GenBank accession number: PQ609686) was compared using BLAST (https://blast.ncbi.nlm.nih.gov/Blast.cgi) (accessed on 31 May 2024) in NCBI, and a neighbor-joining phylogenetic tree was generated using MEGA 11.0 software. ANI values were calculated via the JSpecies Web Server (JSpeciesWS) (http://jspecies.ribohost.com/jspeciesws/) (accessed on 31 May 2024) and compiled into a table. The raw genome sequencing data have been deposited at the NCBI Sequence Read Archive (SRA) database under accession number SRR31647635 and SRR31647636 (https://www.ncbi.nlm.nih.gov/sra/) (accessed on 5 January 2025). The final assembly is available in the GenBank database under accession number CP183925-CP183930.

### 2.3. Extraction and Quantification of CoQ10

This was referred to the method of Chen et al. [28] and slightly modified.

Initially, the cells of YS02 at different growth phases (24 h, 48 h, and 72 h) under four different culture modes were collected into a 100 mL centrifuge tube and then centrifuged at 20,000 rpm for 10 min at 4 °C. The supernatant was discarded. The cell pellet was washed twice with a phosphate buffer solution (1×, pH = 7.4) and centrifuged at 20,000 rpm for 10 min at 4 °C. Next, 1 g of cleaned cells was weighed in a 100 mL centrifuge tube to the nearest 0.05 mg and resuspended in 4 mL of ethanol. The mixture was sonicated and vortexed for 10 min each. Subsequently, 4 mL of n-hexane was added and mixed vigorously for 30 s, sonicated for 10 min, then vortexed for 10 min again. Afterwards, the mixture was centrifuged at 14,000 rpm for 20 min at 4 °C, and the supernatant was collected. The above extraction step was repeated thrice and then concentrated using a rotary evaporator. Lastly, the extract was resuspended in 2 mL of ethanol, filtered through an organic filter membrane (0.22 µm pore size and 13 mm diameter), and stored in the freezer awaiting analysis (−20 °C). All the above samples were prepared in triplicate.

The concentration of CoQ10 was analyzed with a liquid chromatography (Shimadzu LC-20AT, Kyoto, Japan) equipped with binary low-pressure mixing pump (LC-20AT) and a UV-detector (Shimadzu SPD-M20A). The volume of the sample loop is 20 μL. The separation of CoQ10 was performed on a ZORBAX SB-Aq C18 column (4.6 × 150 mm, 5-Micron; Agilent, Santa Clara, CA, USA). The column oven temperature was programmed to 35 °C, the DAD (diode array detector) was set to 275 nm, and the mobile phase consisted of ethanol–methanol (80:20 *v/v*) with a flow rate of 1 mL/min. For the calibration curve, a CoQ10 stock solution was initially prepared at a concentration of 1000 mg/L in ethanol. This stock was then serially diluted to create seven working solutions with concentrations of 1, 10, 50, 100, and 500 mg/L using ethanol as the solvent. Both the stock solution and the working standards were stored at −20 °C for further analysis. CoQ10 was identified by comparing its retention time and ultraviolet spectrogram to those of the standard (studied under the same conditions). Results were reported as mg CoQ10 per kg of sample, with all samples and standards analyzed in triplicate.

### 2.4. Metabolomics Analysis by UPLC-Q-Exactive Orbitrap MS

Under different culture modes (aerobic–dark cultivation and anaerobic–light cultivation), the bacteria were collected at 0 h, 12 h, 24 h, 36 h, 48 h, 60 h, and 72 h. The precipitate (100 mg) after centrifugation was washed twice with 500 μL PBS. It was then frozen in liquid nitrogen, extracted with a mixture of methanol–acetonitrile–water in a ratio of 2:2:1 (total volume 500 μL), vortexed for 30 s, frozen at −20 °C for 30 min, bathed in water at 37 °C for 90 s, and subjected to ultrasonic treatment for 30 s. After 3 cycles of freeze–thaw extraction, the sample was centrifuged at 14,000 rpm for 15 min at 4 °C. The supernatant was collected, dried with nitrogen gas, and redissolved with 100 μL of a solution containing 50% acetonitrile in water. Each sample was filtered through a 0.22 μm organic filter membrane and kept at −20 °C until analysis.

For the UPLC-Q-Exactive Orbitrap MS analysis, a Dionex Ultimate 3000 ultra-high performance liquid chromatography system was coupled with a QE high-resolution mass spectrometer (Thermo Fisher Scientific, Waltham, MA, USA). Chromatographic separation was achieved using an Agilent ZORBAX Eclipse plus RRHD C18 column (150 mm × 3.0 mm, 1.8 µm). The mobile phases included 0.1% formic acid in water (A) and methanol (B). The column temperature was kept at 30 °C, the flow rate was 0.3 mL/min, and the injection volume was 3 µL. The gradient elution program was as follows: 0–1 min, 1% B; 1–5 min, 1–15% B; 5–25 min, 15–99% B; 25–30 min, 99% B; 30–31 min, 99–1% B; 31–35 min, 1% B. The mass spectrometry parameters were set as follows: S-lens RF level: 60; mass range: 100~1500 *m*/*z*; full MS resolution: 70,000; MS/MS resolution: 17,500; normalized collision energy (NCE): 30 eV; spray voltage: 3500 V; sheath gas flow rate: 40; capillary temperature: 320 °C; auxiliary gas heater temperature: 320 °C; auxiliary gas flow rate: 5. Quality control (QC) samples were composed of equal volumes of extracts from all samples. During the analysis, one QC sample was injected after every ten formal samples to assess the stability of the mass spectrometry system throughout the process. Samples were collected in positive (ESI+) and negative (ESI−) switching modes.

### 2.5. Statistical Analysis of Data

For the metabonomic data, the raw data files were loaded into Compound Discoverer 3.3 software (Thermo Fisher Scientific) for ion peak detection, alignment, normalization of peak intensities, and database searches using the *m*/*z* Cloud and ChemSpider databases. The internal Thermo Scientific *m*/*z* Vault spectral library was utilized to facilitate the rapid identification of unknown compounds. The maximum allowable offset time was set at 0.2 min, and the mass tolerance was set to 5 ppm. The signal-to-noise ratio (SNR) threshold was established at 3, with a minimum peak intensity requirement of 1 × 10^6^. Data correction was conducted based on the QC sample peak areas, ensuring that QC coverage was above 50% and the relative standard deviation of QC peak areas remained below 30%.

Afterwards, the data obtained in the above steps were transferred to SIMCA 14.1 software (MKS Umetrics, Malmö, Sweden) for multivariate statistical analysis, which included orthogonal partial least squares discriminant analysis (OPLS-DA) and CV-ANOVA and permutation tests. Potential differential metabolites were screened according to the criteria of variable importance in projection (VIP) > 1, *p* < 0.05, and fold change (FC) > 1.5 or <0.67. To show an overview of relative abundance levels of the identified difference compounds, a heatmap was built using Hiplot (https://hiplot.com.cn/) (accessed on 20 November 2024). Further pathway and enrichment analyses were carried out in MetaboAnalyst v6.0 on-line platform by placing the altered metabolites into their biochemical context.

All samples were duplicated in triplicate, and the results were expressed as means ± standard deviation (Means ± SD). Statistical analysis was carried out using GraphPad Prism 9.5.1 Software (GraphPad Software Inc., San Diego, CA, USA). The IBM SPSS Statistics (version 22.0, SPSS Inc., Chicago, IL, USA) software was adopted for variance analysis (ANOVA). Significance levels were determined by Duncan’s test, with *p* < 0.05 considered statistically significant.

## 3. Results and Discussion

### 3.1. Overview of C. azotoformans-YS02 Genome and Molecular Mechanism for CoQ10 Production

Microbial synthesis is becoming a promising and cost-effective method for CoQ10 production, as microorganisms have high CoQ10 content and are easily cultivated [29]. After multiple rounds of enrichment and separation, a novel strain, designated YS02, was isolated for its ability to produce CoQ10. The strain YS02 was identified as a gram-negative bacteria with an oval shape (Figure S1B). On the nutrient agar plate, it formed dark red, round colonies that were well defined, smooth in texture, and moist in appearance (Figure S1A). To genetically characterize the isolate strain YS02 and ascertain the molecular basis of CoQ10 formation, we analyzed the whole genome of YS02 through genome sequencing, assembly, and annotation.

**Figure 1:**
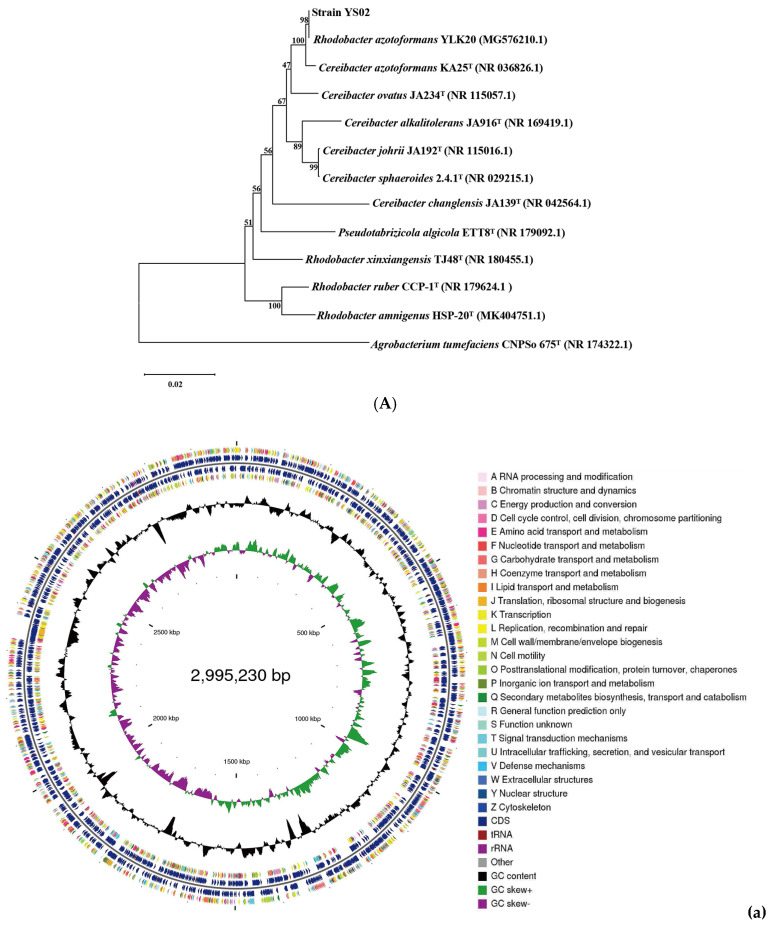

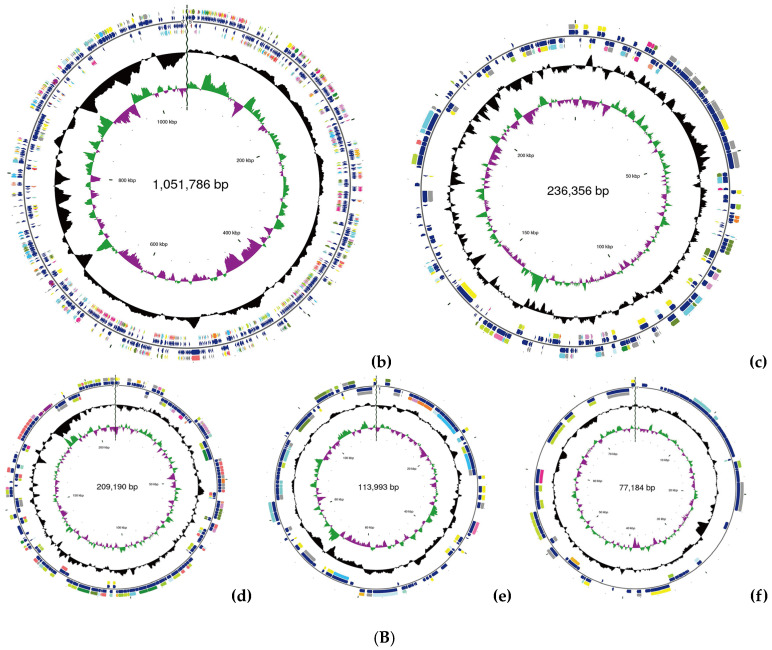
(**A**) Phylogenetic analysis of strain YS02 based on 16S rRNA sequences was performed using the neighbor-joining approach. (**B**). Circular genome representation, including the following: (**a**) chromosome, (**b**) plasmid A, (**c**) plasmid B, (**d**) plasmid C, (**e**) plasmid D, and (**f**) plasmid E. Features are displayed from the outermost to the innermost circles as follows: coding sequences (CDSs), color-coded according to clusters of orthologous groups (COG) functional categories, on the forward strand; tRNA and rRNA genes on both the forward and reverse strands; CDSs on the reverse strand; GC content (depicted as deviations from the average GC content of the entire sequence; positive values plotted outward and negative values inward); GC skew (G − /G + C), where the leading and lagging strands can be inferred based on GC skew values (typically, GC skew > 0 for the leading strand and GC skew < 0 for the lagging strand). The innermost circle indicates genome size.

The phylogenetic tree constructed by using 16S rRNA gene sequences is presented in Figure 1A. Homologous sequence comparison was performed using the blastn tool (https://blast.ncbi.nlm.nih.gov/Blast.cgi) (accessed on 31 May 2024) in the NCBI database. The analysis revealed that the 16S rRNA gene of YS02 had a 100% sequence similarity with *C. azotoformans* KA25 and 98% similarity with *R. azotoformans* YLK20. Additionally, ANI values are used to measure overall similarity between genome sequences. According to the whole genome sequence of YS02, 12 representative strains were selected from the NCBI database and reference database GenomesDB. Phylogenetic analysis and classified identification of YS02 were further carried out by calculating the ANIb values (Table S1). The results disclosed that strain YS02 had 98.4% (highest) similarity with the *C. azotoformans* KA25 and 98.18% (second highest) similarity with *C. azotoformans* YLK20 (Table S1). Additionally, a 95–96% cut-off was proposed for species demarcation ANI. Phylogenetic systematics of new genome scale data classified *Rhodobacter* as a sister group to *Cereibacter*, making *R. azotoformans* synonymous and interchangeable with *C. azotoformans* [30,31]. Since the ANI value is higher than 96%, the isolated strain was ultimately designated as *C. azotoformans-*YS02.

The general features of the YS02 genome are summarized in Table 1. Following quality control, the total number of bases was 1217,884,848 base pairs (bp), with a Q30 base percentage exceeding 94.98%. The length of the largest reads was 24,752 bp, and the reads’ N50 was 9417 bp. The clean data after quality control underwent de novo assembly to obtain the genome sequence. Then, gene prediction was performed on the assembled sequence. A complete chromosome and five plasmids were obtained through the assembly process. The CGView genome map of YS02 can be seen in Figure 1B. The genome of YS02 spans 4,683,739 bp with a GC content of 68.02%. It comprises a 2,995,230 bp chromosome and five plasmids: plasmid A (1,051,786 bp), plasmid B (236,356 bp), plasmid C (209,190 bp), plasmid D (113,993 bp), and plasmid E (77,184 bp). The plasmid classification was based on structural features and a lack of essential housekeeping genes. A total of 4432 protein coding genes were predicted with sequence lengths of 4,071,321 bp, giving a coding intensity of 86.92%. The prediction results revealed 12 rRNA genes, 55 tRNA genes, and 33 sRNA in the genome.

**Figure 2:**
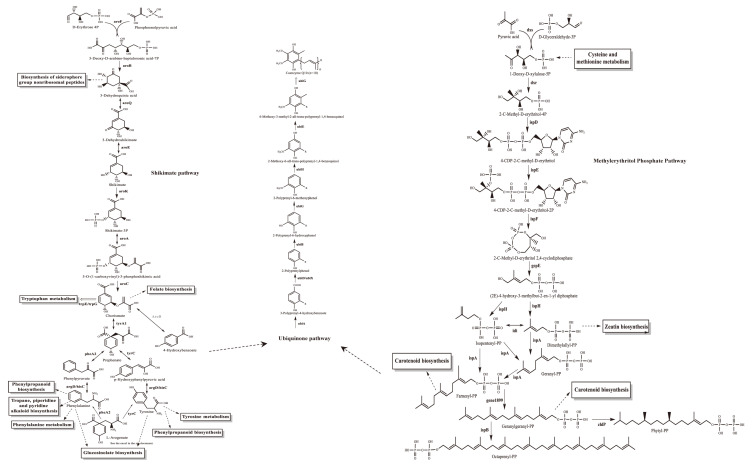
The CoQ10 rough generation pathway in C. azotoformans-YS02 was obtained through WGS analysis. Notes: the solid line indicates the one-step reaction, the dotted arrows indicate omitted intermediate multi-step reactions, the bidirectional arrows indicate reversible reactions, the double-line arrows indicate omitted multi-step reaction and marks the annotated relevant enzyme, the annotated homologous proteins are marked in gray font.

The gene annotation for YS02 is based on the Non-Redundant Protein Sequence (NR) database, Swiss-Prot, Pfam, Clusters of Orthologous Groups (COG), Gene Ontology (GO), and the Kyoto Encyclopedia of Genes and Genomes (KEGG) database. The summary information is displayed in Table 1 and Table S2, respectively. Among them, 3571 genes were assigned to 23 different types in the COG categories, reflecting the organism’s efficiency in energy production and conversion, amino acid transport and metabolism and secondary metabolites biosynthesis, and transport and catabolism (Figure S2A). Additionally, the main biosynthesis pathways relevant to CoQ10, including the shikimate pathway, the ubiquinone biosynthesis pathway, and the methylerythritol phosphate (MEP) pathway [29,32], were identified within the amino acid metabolism, metabolism of cofactors and vitamins, and metabolism of terpenoids and polyketides pathways, respectively (Figures S2B and S3). Based on both structural and functional gene annotation information, we obtained the rough pathway for CoQ10 biosynthesis in YS02 at the molecular level (Figure 2). As summarized in Table 2, the major target enzymes involved in the prominent coenzyme Q10 biosynthesis pathway [21,32], such as DXS, DXR, Idi, IspB, UbiA, UbiE, UbiG, were also annotated. The detailed annotation information of enzymes in YS02 can be seen in Table S6. These demonstrated that YS02 has a good molecular foundation for the production of CoQ10.

### 3.2. Determination of CoQ10 in C. azotoformans-YS02 Under Four Different Culture Modes

It has been reported that PNSB are desirable and preferred among microorganisms capable of producing CoQ10 [21,29]. Although *R. sphaeroides* has been discovered with a good ability to produce CoQ10, these strains still require light and anaerobic conditions to produce higher CoQ10 titers in practical production [21,28]. In order to assess the production of CoQ10 in *C. azotoformans*-YS02 in the present work, the concentration of CoQ10 in YS02 was quantitatively analyzed utilizing the developed liquid chromatography.

**Figure 3:**
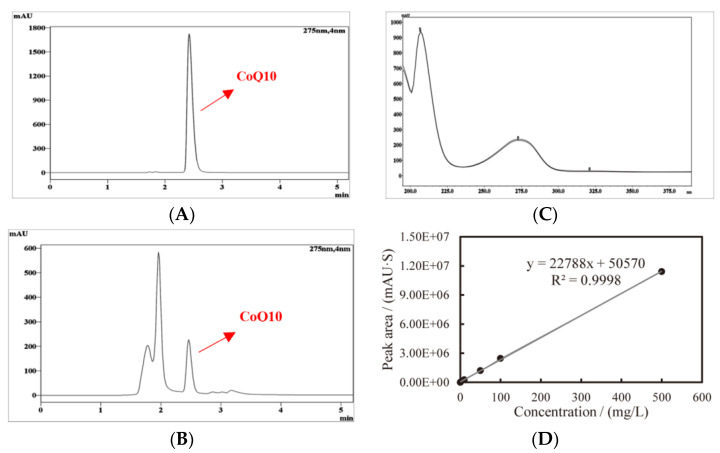
Typical HPLC chromatogram and ultraviolet spectrogram of CoQ10 acquired at 275 nm and the constructed standard curve, (**A**) CoQ10 standard, (**B**) CoQ10 in sample, (**C**) comparison spectrogram of standard and sample, and (**D**) standard curve of CoQ10.

The typical chromatogram of the CoQ10 standard, YS02 extract, and the overlay of their ultraviolet spectrogram are reflected in Figure 3. To determine the CoQ10 in YS02, a calibration curve was established using the external standard method, and the linearity was found to be 0.9998 over the range of 1 mg/L–500 mg/L (Figure 3D). Meanwhile, growth of YS02 cells was detected by measuring the optical density at 600 nm (OD_600_). Through quantifying the concentration of CoQ10 in YS02 cells at 0 h, 24 h, 48 h, and 72 h under four culture modes (anaerobic–light, 24 h of anaerobic–light followed by 48 h of aerobic–dark, aerobic–dark, and 24 h of aerobic–dark followed by 48 h of anaerobic–light) to probe into the growth strategy that makes its CoQ10 production competitive with regard to fermentation research applications.

**Figure 4:**
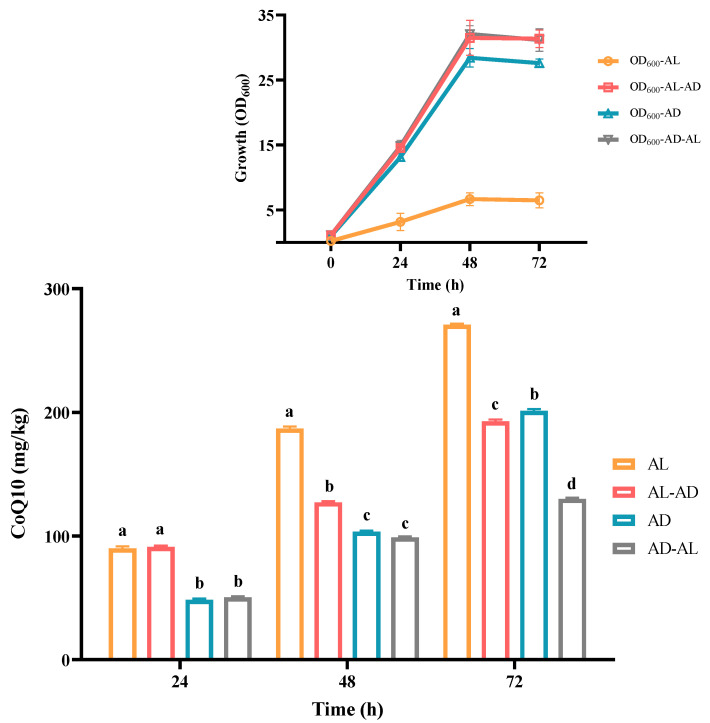
The concentration of CoQ10 in *C. azotoformans*-YS02 and the growth of YS02 cells under four different culture modes (n = 6). Data in the figure are the mean ± SD. Different lowercase letters denote significance (*p* < 0.05). Notes: AL: anaerobic–light; AD: aerobic–dark. AL-AD: 24 h of AL followed by 48 h of AD; AD-AL: 24 h of AD followed by 48 h of AL.

As is shown in Figure 4, *C. azotoformans* survived in an anaerobic environment with the support of light energy, and the content of CoQ10 was found to be about 270 mg/kg. Compared with common food (mainly fish visceral tissue and meat and vegetables, 0.4–130 mg/kg) [33], its content is more than 2~500 fold. So, YS02 can be used as an optional source of CoQ10. Although a good concentration of CoQ10 was achieved, cell growth of YS02 remained notably low (the value of OD_600_ is only about 5). This may be attributed to the light shading effect, which is similar to that observed in *R. sphaeroides* [28]. In terms of energy consumption, the faster growth rate and higher biomass of microorganisms are advantageous for industrial-scale applications. Hence, this method is not competitive in substance mass production. In view of the above situation, we adopted two strategies: (1) anaerobic–light culture for 24 h following aerobic–dark culture for 48 h and (2) aerobic–dark culture for 24 h following anaerobic–light culture for 48 h. However, there was no high CoQ10 content achieved by employing the mixed culture modes except for the obvious increase in the biomass of YS02 cells (Figure 4). Importantly, in Figure 4, compared with anaerobic–light cultivation, we found that YS02 demonstrates better cell biomass in the aerobic–dark condition. Moreover, the content of CoQ10 reached 201 mg/kg, which is higher than the above two mixed culture modes. Therefore, it was recommended that the aerobic–dark cultivation could be employed to produce CoQ10 using YS02 in future research applications.

### 3.3. Analysis of Differential Metabolites in C. azotoformans-YS02 Under Different Culture Conditions

**Figure 5:**
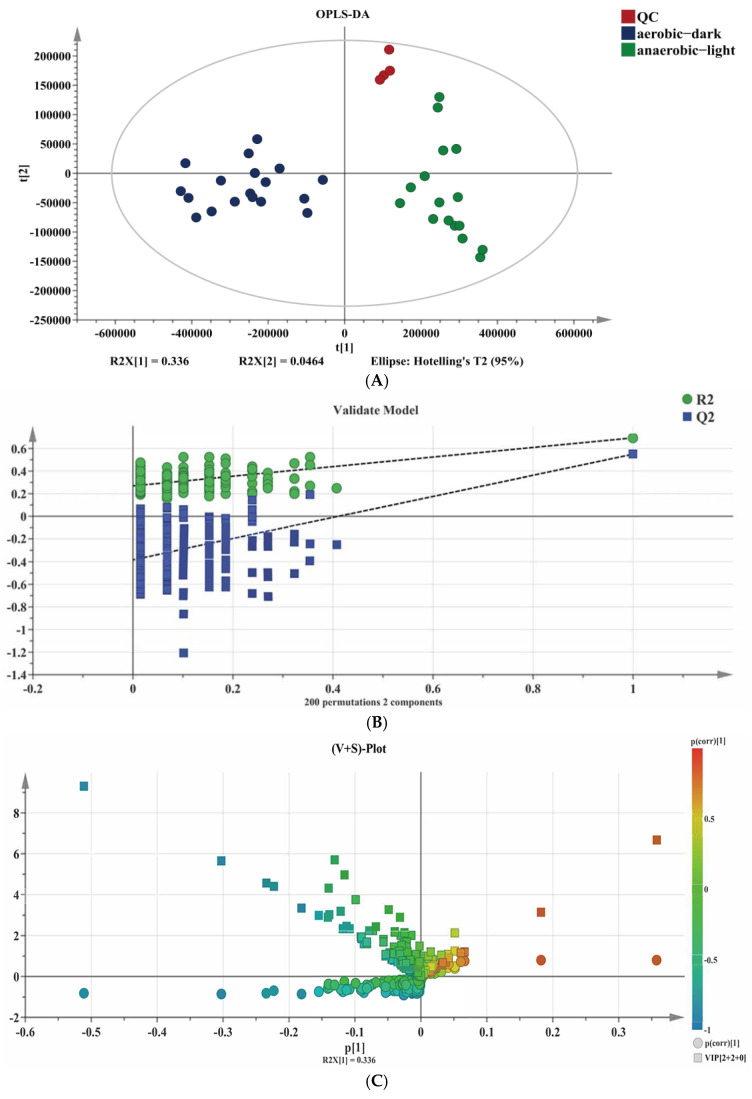
Combined chemometrics and UPLC-Q-Exactive Orbitrap MS-based untargeted analysis for difference metabolites’ identification. (**A**) Scatter plot of OPLS-DA scores, (**B**) corresponding validated model, and (**C**) (V + S)-plot.

Metabolites are the end-products or intermediates of metabolic processes that have the role of modulating the phenotype of biological systems [34]. For the sake of delving deeper into the reason for the metabolic difference of YS02 under both anaerobic–light and aerobic–dark culture conditions, we applied an untargeted metabolism approach to screen for compounds and analyze the differential small molecular metabolites in YS02. To compare the profile of small molecule metabolites from YS02 under anaerobic–light and aerobic–dark cultivation, we obtained MS data using UPLC-Q-Exactive Orbitrap MS. Among these samples, 542 molecular features were obtained after the pre-processing of raw data and sufficient separation of anaerobic–light and aerobic–dark samples was achieved in the OPLS-DA results (Figure 5A), indicating a clear difference in their metabolic profiles. The CV-ANOVA verification (Table S3) manifested in the OPLS-DA model had statistical significance (*p* < 0.05). To verify the predictability of the OPLS-DA model, 200 permutations were conducted (Figure 5B). The results show that the original R^2^ and Q^2^ (the two rightmost points) are both larger than the corresponding value after permutation (the left scatter points), and the intercept of R^2^ does not exceed 0.3 (0.27), and the intercept of Q^2^ does not exceed 0.05 (−0.383). This suggests that there is no overfitting for the experiment; the OPLS-DA model is reliable and can be effectively utilized for further analysis.

**Figure 6:**
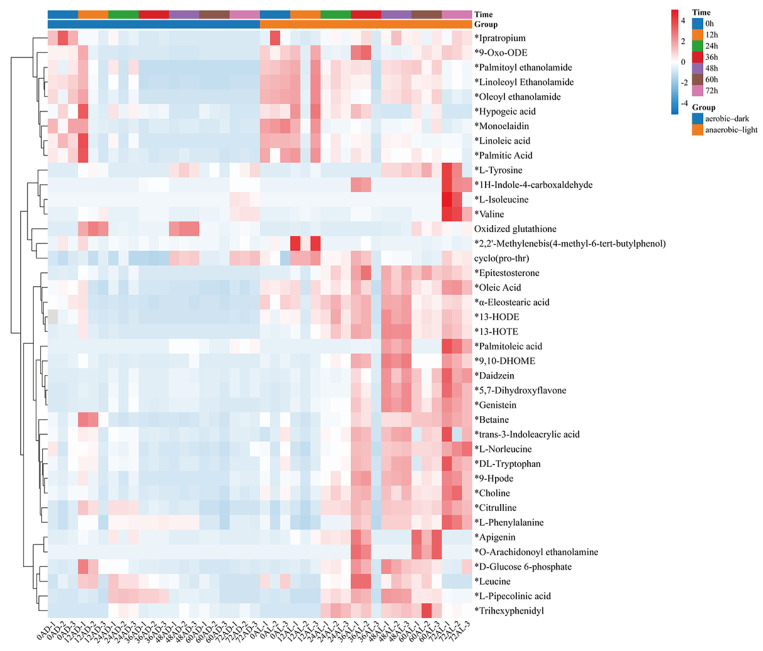
Heatmap of identified differential compounds in YS02 samples. The color scale represents the scaled abundance of each compound with red indicating higher abundance and blue indicating lower abundance (*p* < 0.05). The 38 significantly up-regulated compounds (VIP score > 1 and FC > 1.5) are indicated by asterisks (*); the 2 significantly down-regulated compounds (VIP score > 1 and FC < 0.67) are without asterisks. Notes: AL: anaerobic–light; AD: aerobic–dark.

The distribution of the identified 542 metabolites from anaerobic–light and aerobic–dark samples was showcased in a (V + S)-plot (Figure 5C), which serves to visualize the *p* values, *p* correlation coefficients (*p*(corr)), and VIP scores. The VIP scores, which are a hallmark of a high discrimination potential, were determined by the OPLS-DA model. Compared in the HMDB database, a total of 40 compounds were ultimately found to have a VIP score > 1, *p* < 0.05, and FC > 1.5 or < 0.67 from the anaerobic–light and aerobic–dark groups. Among them, 38 compounds in the anaerobic–light group were significantly up-regulated compared with those in the aerobic dark group, while 2 compounds were significantly down-regulated (Figure 6). Corresponding values for these differential compounds are listed in Table S4. The primary classification of 40 differential compounds mainly belongs to carboxylic acids and derivatives, fatty Acyls, flavonoids, indoles and derivatives, organonitrogen compounds, isoflavonoids, and organooxygen compounds. The analysis indicated that, although the cell growth of YS02 under anaerobic–light conditions was weaker than under aerobic–dark conditions (Figure 4), the abundance of its small molecular metabolites demonstrated a notable advantage (Figure 6). This disparity may account for its elevated production of CoQ10.

### 3.4. Enrichment Analysis and Pathway Analysis Based on Differential Metabolites in C. azotoformans-YS02

**Figure 7:**
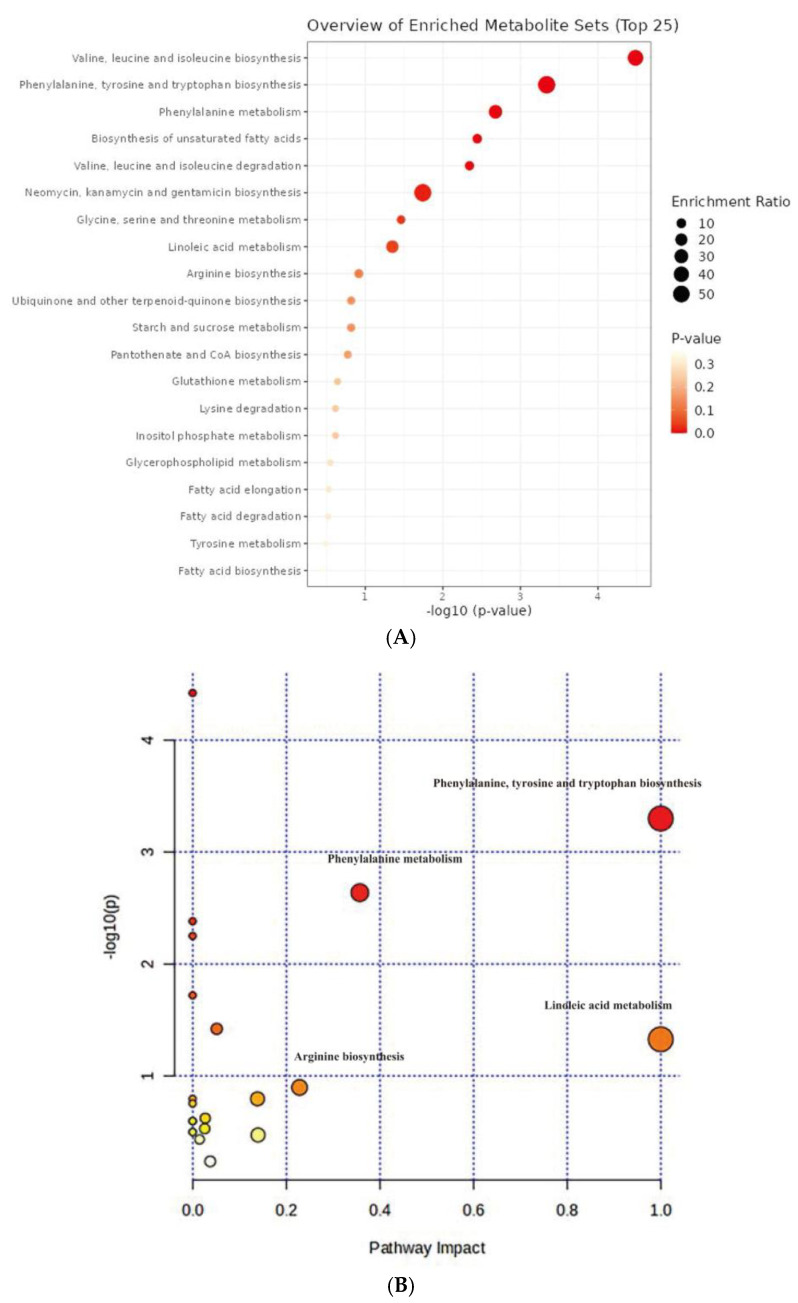
Analysis of metabolic pathway based on KEGG database in *C. azotoformans*-YS02. (**A**) Enrichment analysis results of metabolites set; (**B**) pathway enrichment analysis of metabolites set.

Considering the observed differences in CoQ10 production under both anaerobic–light and aerobic–dark culture conditions, we sought to conduct enrichment scoring and pathway analysis of 40 differential metabolites to elucidate the causes. The purpose was to find the advantageous metabolic pathway under the anaerobic–light condition in order to find a possible target for modifying the metabolic pathway under the aerobic–dark condition, thereby enhancing the production of CoQ10. Metabolic pathway analyses were performed by using MetaboAnalystR, applying the hypergeometric algorithm for over representation analysis and the relative-betweenness centrality algorithm for pathway topology analysis (Figure 7A,B).

The top 25 significantly enriched metabolic pathways are displayed in Figure 7A. All of the 20 metabolic pathways show certain significance, and the enrichment ratio ranges from 0 to 1. According to the comprehensive assessment of the size and color of the dots, we discovered that valine, leucine, and isoleucine biosynthesis (leucine, isoleucine, and L-valine) and phenylalanine, tyrosine, and tryptophan biosynthesis (phenylalanine and L-tyrosine) are the top two signal pathways in terms of enrichment degree, with a smaller *p*-value and a larger enrichment ratio (Figure 7A). Pathway analysis referencing the KEGG pathway database revealed that there were significant differences in 4 out of 21 metabolic pathways in YS02 under different culture modes (Figure 7B), primarily including phenylalanine, tyrosine, and tryptophan biosynthesis, phenylalanine metabolism (phenylalanine and L-tyrosine), linoleic acid metabolism (linoleic acid), and arginine biosynthesis (citrulline) (pathway impact > 0.2). Within this, phenylalanine, tyrosine, and tryptophan biosynthesis ranked first in terms of significance and pathway influence (*p* = 0.000503, −log10(*p*) = 3.30, and impact = 1.0), suggesting that this pathway plays an important part in the regulation of the overall metabolic network. Despite the relatively low degree of influence and significance of the other pathways depicted in Figure 7B, they still hint at potential functions in specific biological processes.

It is worth noting that the ubiquinone and other terpenoid–quinone biosynthesis (L-tyrosine) signaling pathway had also been found significant (*p* = 0.15902, and impact = 0.0) (Figure 7B and Table S5). These were pivotal pathways for CoQ10 synthesis in YS02. In our genome annotation analysis (Figure S3 and Table 2), we annotated correlated enzyme genes for synthesizing CoQ10. Some key enzymes, namely UbiA, UbiD, UbiG, UbiH, and UbiE, involved in CoQ10 synthesis [35,36,37] were annotated in the pathway of ubiquinone and other terpenoid–quinone biosynthesis (Figure S3C and Table 2). In addition, as shown in Figure S3C, phenylalanine, tyrosine, and tryptophan biosynthesis is an upstream pathway for CoQ10 synthesis, closely connected to 4-hydroxybenzoate, a key precursor in CoQ10 production [38]. Therefore, despite the production of CoQ10 being regulated by multiple metabolic pathways [32], we hypothesized that the phenylalanine, tyrosine, and tryptophan biosynthesis pathway might explain the variation in CoQ10 content observed in YS02 under anaerobic–light and aerobic–dark conditions. Although UbiC, the enzyme that connects metabolic pathways, was not annotated in YS02 (Figure S3C), we found its homologous protein, ArcB (gene 0258) (Table S6). In some previous studies, XanB2 was proved to be a bifunctional chorismate lyase that hydrolyzes chorismate, the end product of the shikimate pathway, to produce 3-hydroxybenzoic acid (3-HBA) and 4-hydroxybenzoic acid (4-HBA) [39]. The protein ArcB in *Azoarcus* sp. BH72 was confirmed to possess the same enzymatic function as XanB2 [40]. Therefore, ArcB may serve as an alternative source of 4-HBA for CoQ10 biosynthesis in YS02. However, the detailed mechanism of our hypothesis still needs in-depth investigation. It is well known that use of genome-scale metabolic models allows for the prediction of the effect of gene manipulations on metabolic pathways [41]. We propose that follow-up research could set out from the upstream pathway of CoQ10 synthesis. By applying genetic modification or metabolic engineering techniques, it is possible to enhance metabolic flux during aerobic–dark cultivation, leading to further improvements in CoQ10 production by YS02 for industrial scale application.

## 4. Conclusions

Our work offers the novel strain *C. azotoformans*-YS02 as a promising platform for CoQ10 production. Genome analysis suggested its molecular potential for CoQ10 biosynthesis. Through gene annotation, we obtained the preliminary biosynthetic pathway of CoQ10 in YS02. The aerobic–dark fermentation was identified as the optimal cultivation method for subsequent application. Metabolomics analysis highlighted the importance of phenylalanine, tyrosine, and tryptophan biosynthesis in regulating CoQ10 production. These outcomes advance our understanding of CoQ10 production and pave the way for further optimization of YS02 for industrial applications. Metabolic engineering techniques can be employed to augment the activities of key enzymes involved in the biosynthetic pathway, leading to increased CoQ10 yields. Furthermore, this novel strain may also serve as a platform for producing other valuable bioactive compounds through targeted metabolic engineering. The fundamental understanding gained from this research can further accelerate its basic molecular research and biotechnological applications.

## Figures and Tables

**Table 1:** General features of *C. azotoformans*-YS02 genome.

Parameters	Values
Clean bases	1,217,884,848 bp
Clean Q30	94.98%
largest reads length	24,752 bp
Reads N50	9417 bp
Chromosome	1
Plasmid	5
Genome Size	4,683,739 bp
G + C	68.02%
Coding gene	4432
Gene total length	4,071,321 bp
Gene average length	918.62 bp
Gene/Genome	86.92%
tRNA	55
rRNA	12
sRNA	33
Repeat DNAs	40
SINEs ^a^	16
LINEs ^b^	8
Genes of KEGG	3252
Genes of COG	3571

^a^ SINEs: short interspersed nuclear elements. ^b^ LINEs: long interspersed nuclear elements.

**Table 2:** The summary of gene annotation information of important enzymes involved in CoQ10 biosynthesis.

Gene ID	Gene Name	Location	Length (bp)	NR Description	Swiss-Prot Description	Pfam ID	COG ID	GO ID	KO ID	**KO Description**
gene1887	*dxs*	Chromosome (1,871,388–1,869,442)	1947	MULTISPECIES: 1-deoxy-D-xylulose-5-phosphate synthase	1-deoxy-D-xylulose-5-phosphate synthase 1 OS = Cereibacter sphaeroides (strain ATCC 17023/DSM 158/JCM 6121/CCUG 31486/LMG 2827/NBRC 12203/NCIMB 8253/ATH 2.4.1.) OX = 272,943 GN = dxs1 PE = 3 SV = 1	PF13292; PF02779; PF02780	COG1154	GO: 0016114; GO: 0009228;GO: 0052865; GO: 0000287; GO: 0030976;GO: 0008661	K01662	1-deoxy-D-xylulose-5-phosphate synthase [EC: 2.2.1.7]
gene1977	*dxr*	Chromosome (1,960,897–1,959,713)	1185	1-deoxy-D-xylulose-5-phosphate reductoisomerase	1-deoxy-D-xylulose 5-phosphate reductoisomerase OS = Cereibacter sphaeroides (strain ATCC 17025/ATH 2.4.3) OX = 349,102 GN = dxr PE = 3 SV = 1	PF08436; PF02670; PF13288	COG0743	-	K00099	1-deoxy-D-xylulose-5-phosphate reductoisomerase [EC: 1.1.1.267]
gene2789	*dxs*	Chromosome (2,788,800–2,786,887)	1914	MULTISPECIES: 1-deoxy-D-xylulose-5-phosphate synthase	1-deoxy-D-xylulose-5-phosphate synthase 2 OS = Cereibacter sphaeroides (strain ATCC 17023/DSM 158/JCM 6121/CCUG 31486/LMG 2827/NBRC 12203/NCIMB 8253/ATH 2.4.1.) OX = 272,943 GN = dxs2 PE = 3 SV = 1	PF13292; PF02779; PF02780	COG1154	GO: 0016114; GO: 0009228; GO: 0052865; GO: 0000287; GO: 0030976; GO: 0008661	K01662	1-deoxy-D-xylulose-5-phosphate synthase [EC: 2.2.1.7]
gene0908	*idi*	Chromosome (915,412–915,945)	534	isopentenyl-diphosphate Delta-isomerase	Isopentenyl-diphosphate Delta-isomerase OS = Cereibacter sphaeroides (strain ATCC 17025/ATH 2.4.3) OX = 349,102 GN = idi PE = 3 SV = 2	PF00293	COG1443	-	K01823	isopentenyl-diphosphate Delta-isomerase [EC:5.3.3.2]
gene2309	*ispB*	Chromosome (2,300,848–2,301,849)	1002	MULTISPECIES: polyprenyl synthetase family protein	Octaprenyl diphosphate synthase OS = Shigella flexneri OX = 623 GN = ispB PE = 3 SV = 1	PF00348	COG0142	GO: 0008299; GO: 0016740	K02523	octaprenyl-diphosphate synthase [EC: 2.5.1.90]
gene0203	*ubiA*	Chromosome (198,354–199,346)	993	4-hydroxybenzoate octaprenyltransferase	4-hydroxybenzoate polyprenyltransferase, mitochondrial OS = Dictyostelium discoideum OX = 44,689 GN = coq2 PE = 3 SV = 1	PF01040	COG0382	-	K03179	4-hydroxybenzoate polyprenyltransferase [EC: 2.5.1.39]
gene2225	*ubiD*	Chromosome (2,217,921–2,219,414)	1494	MULTISPECIES: UbiD family decarboxylase	3-octaprenyl-4-hydroxybenzoate carboxy-lyase OS = Pseudomonas syringae pv. syringae (strain B728a) OX = 205,918 GN = ubiD PE = 3 SV = 2	PF01977; PF20696; PF20695	COG0043	-	K03182	4-hydroxy-3-polyprenylbenzoate decarboxylase [EC: 4.1.1.98]
gene2993	*ubiE*	Chromosome (2,992,547–2,991,795)	753	MULTISPECIES: bifunctional demethylmenaquinone methyltransferase/2-methoxy-6-polyprenyl-1,4-benzoquinol methylase UbiE	Ubiquinone/menaquinone biosynthesis C-methyltransferase UbiE OS = Cereibacter sphaeroides (strain ATCC 17025/ATH 2.4.3) OX = 349,102 GN = ubiE PE = 3 SV = 1	PF01209; PF13649; PF08241; PF13847; PF08242; PF13489; PF05175	COG2226	GO: 0032259; GO: 0006744; GO: 0009060; GO: 0009234; GO: 0043770; GO: 0102955; GO: 0043333; GO: 0102027; GO: 0102094	K03183	demethylmenaquinone methyltransferase/2-methoxy-6-polyprenyl-1,4-benzoquinol methylase [EC: 2.1.1.163 2.1.1.201]
gene2814	*ubiG*	Chromosome (2,811,344–2,810,601)	744	MULTISPECIES: bifunctional 2-polyprenyl-6-hydroxyphenol methylase/3-demethylubiquinol 3-O-methyltransferase UbiG	Ubiquinone biosynthesis O-methyltransferase OS = Cereibacter sphaeroides (strain ATCC 17025/ATH 2.4.3) OX = 349,102 GN = ubiG PE = 3 SV = 1	PF13489; PF08241; PF13649; PF13847; PF08242; PF02353; PF08003; PF06325; PF01209	COG2227	GO: 0032259; GO: 0006744; GO: 0005737; GO: 0102208; GO: 0008425; GO: 0008689	K00568	2-polyprenyl-6-hydroxyphenyl methylase/3-demethylubiquinone-9 3-methyltransferase [EC: 2.1.1.222 2.1.1.64]
gene0627	*ubiH*	Chromosome (626,189–624,990)	1200	MULTISPECIES: UbiH/UbiF family hydroxylase	Ubiquinone hydroxylase UbiL OS = Rhodospirillum rubrum (strain ATCC 11170/ATH 1.1.1/DSM 467/LMG 4362/NCIMB 8255/S1) OX = 269,796 GN = ubiL PE = 1 SV = 1	PF01494; PF00890	COG0654	GO: 0006744; GO: 0071949; GO: 0016705	K03185	2-octaprenyl-6-methoxyphenol hydroxylase [EC: 1.14.13.-]
gene2678	*ubiH*	Chromosome (2,676,978–2,678,204)	1227	UbiH/UbiF/VisC/COQ6 family ubiquinone biosynthesis hydroxylase	Ubiquinone hydroxylase UbiL OS = Rhodospirillum rubrum (strain ATCC 11170/ATH 1.1.1/DSM 467/LMG 4362/NCIMB 8255/S1) OX = 269,796 GN = ubiL PE = 1 SV = 1	PF01494	COG0654	-	K03185	2-octaprenyl-6-methoxyphenol hydroxylase [EC: 1.14.13.-]

## Data Availability

The raw genome sequencing data have been deposited at the NCBI Sequence Read Archive (SRA) database under accession number SRR31647635 and SRR31647636, and the other data will be made available on request.

## Supplementary Materials

The following supporting information can be downloaded at https://www.mdpi.com/article/10.3390/antiox14040429/s1: Figure S1: The morphological features of *Cereibacter azotoformans*-YS02 colonies on agar medium and the gram staining micrograph, (A) Colony of isolate YS02 on nutrient agar plate, (B) Gram staining micrograph of isolate YS02; Table S1: ANIb summary for Strain YS02; Table S2: The annotation genes statistics of *Cereibacter azotoformans*-YS02 in different databases; Figure S2: Classified statistical histogram of gene annotation of *Cereibacter azotoformans*-YS02, (A) COG function classification, (B) Histogram of KEGG; Table S3: CV-ANOVA results; Table S4: The detailed information of 40 differential compounds; Figure S3: The information of gene annotation pathways in KEGG database related to Coenzyme Q10 biosynthesis, (A) Gene annotation in phenylalanine, tyrosine, and tryptophan biosynthesis pathway, (B) Gene annotation in terpenoid backbone biosynthesis pathway, (C) Gene annotation in the ubiquinone and other terpenoid–quinone biosynthesis pathway (phenylalanine, tyrosine, and tryptophan biosynthesis was marked with a red box); Table S5: The results of pathway analysis. Table S6: The detailed gene annotation information of strain YS02.
