# Supplementary material for: Detection of Cereibacter azotoformans-YS02 as a Novel Source of Coenzyme Q10 and Its Metabolic Analysis
Source: Antioxidants (Basel). 2025 Apr 1;14(4):429. doi: 10.3390/antiox14040429 (PMC12024278; doi:10.3390/antiox14040429)
Supplement: Supplementary file 1 [file antioxidants-14-00429-s001.zip › Supplementary material.pdf]

Supplementary materials for

Detection of *Cereibacter azotoformans*-YS02 as a Novel Source of Coenzyme Q10 and Its Metabolic Analysis

=

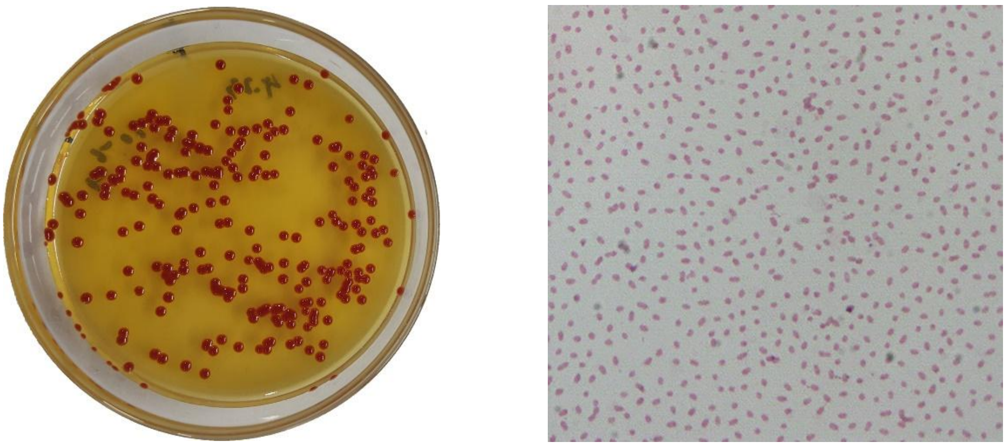

A B

Figure S1. The morphological features of *Cereibacter azotoformans*-YS02 colonies on agar medium and the gram staining micrograph, A Colony of isolate YS02 on nutrient agar plate, B Gram staining micrograph of isolate YS02.

Table S1. ANIb summary of Strain YS02.

**Genome**	**ANIb (%)**	**Aligned (%)**	**Aligned (bp)**	**Total (bp)**
Cereibacter azotoformans KA25 [T]	98.4	82.87	3881499	4683739
Cereibacter azotoformans YLK20	98.18	81.18	3802305	4683739
Cereibacter sediminicola JA983 [T]	94.86	76.96	3604601	4683739
Cereibacter johrii JA192 [T]	85.19	61.53	2882102	4683739
Cereibacter sphaeroides 2.4.1 [T]	85.12	64.13	3003501	4683739
Cereibacter sphaeroides KD131 KD131; KCTC 12085	84.89	63.45	2971681	4683739
Cereibacter ovatus JA234 [T]	80.93	52.38	2453246	4683739
Cereibacter changlensis JA139 [T]	77.89	50.09	2345901	4683739
Rhodobacter amnigenus HSP-20 [T]	74.86	36.59	1713614	4683739
Rhodobacter ruber CCP-1 [T]	74	36.52	1710688	4683739
Pseudotabrizicola algicola ETT8 [T]	73.87	37.69	1765336	4683739
Agrobacterium tumefaciens CNPSo 675 [T]	67.05	17.54	821506	4683739

Table S2. The annotation genes statistics of *Cereibacter azotoformans*-YS02 in different databases.

**Database name**	NR	Swiss-Prot	Pfam	GO
**Gene No.**	4402	3110	3678	1720

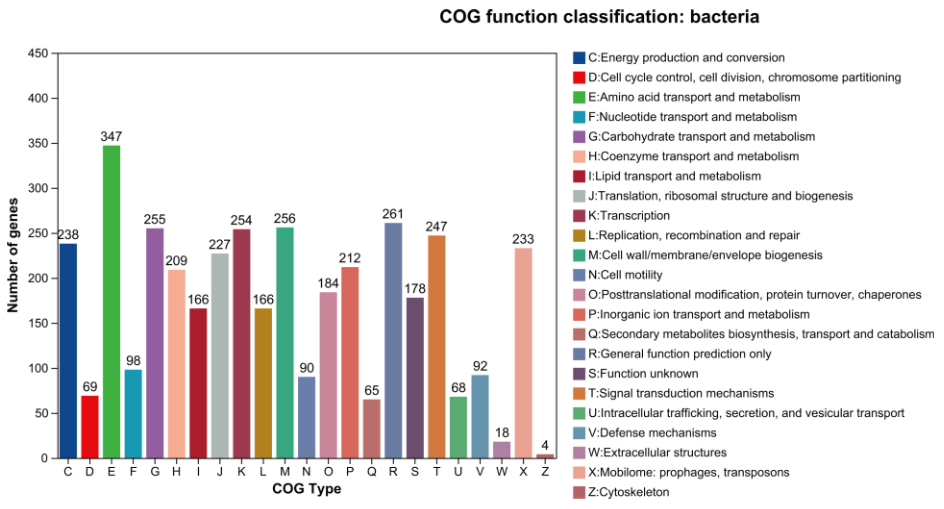

A

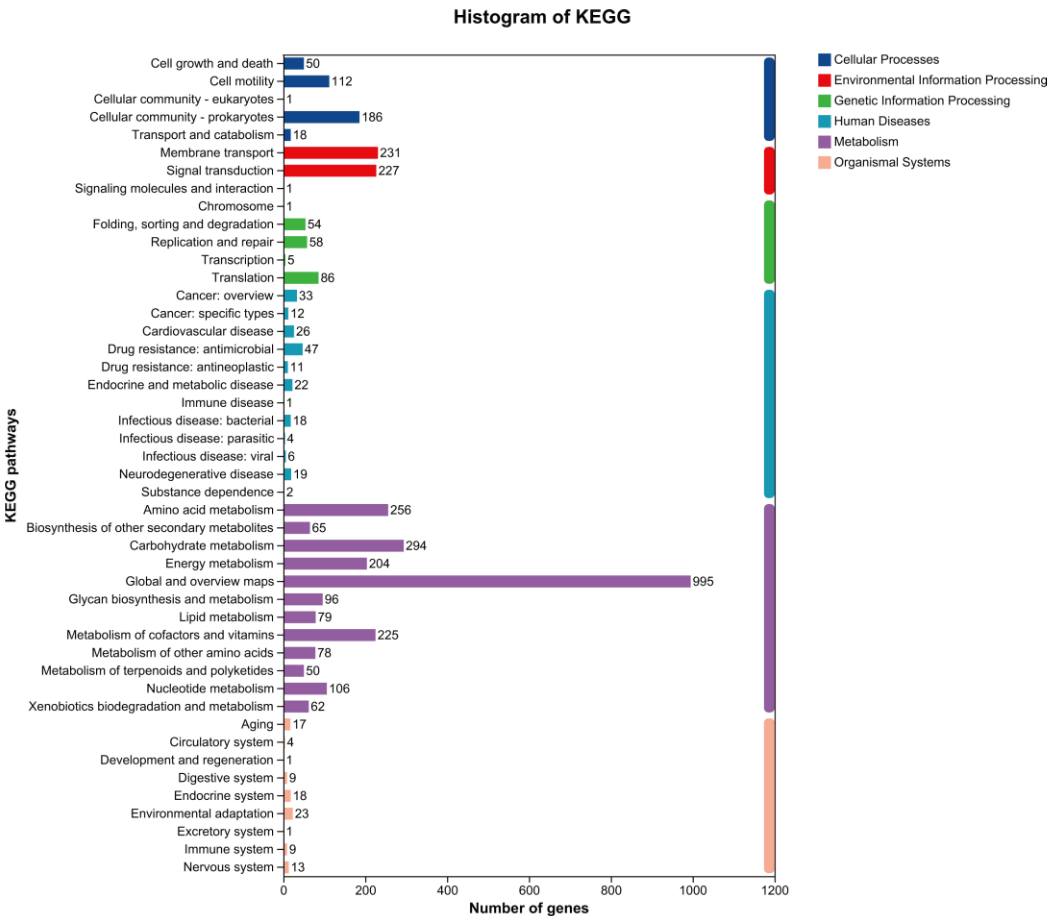

B

Figure S2. Classified statistical histogram of gene annotation of *Cereibacter azotoformans*-YS02, **A** COG function classification, **B** Histogram of KEGG.

Table S3. CV-ANOVA results.

CV-ANOVA	SS	DF	MS	F	p	SD
**Total corr.**	94	94	1			1
**Regression**	50.4015	16	3.1501	5.63569	7.77E-08	1.77485
**Residual**	43.5985	78	0.558955			0.747633

Table S4. The detailed information of 40 differential compounds.

Name	m/z	adduct	Adduct (m/z)	ID	Formula	Class	Sub Class	p[1]	VIP[2+2+0]	FC
**2,2'-Methylenebis(4-methyl-6-tert-butylphenol)**	339.23158	M-H	339.233	HMDB0244434	C23H32O2	Benzene and substituted derivatives	Diphenylmethanes	-0.0260326	1.19538	2.101947665
**Palmitoyl ethanolamide**	322.27254	M+Na	322.2716	HMDB0002100	C18H37NO2	Carboximidic acids and derivatives	Carboximidic acids	-0.112886	2.45021	3.068288707
**Oxidized glutathione**	307.08429	M+2H	307.0833	HMDB0003337	C20H32N6O12S2	Carboxylic acids and derivatives	Amino acids, peptides, and analogues	0.0116543	1.00677	0.299159649
**cyclo(pro-thr)**	199.10827	M+H	199.1077	HMDB0341303	C9H14N2O3	Carboxylic acids and derivatives	Amino acids, peptides, and analogues	0.0409074	1.06376	0.6396926
**L-Pipecolinic acid**	147.11329	M+NH4	147.1128	HMDB0000716	C6H11NO2	Carboxylic acids and derivatives	Amino acids, peptides, and analogues	-0.0220518	1.46255	1.556543938
**L-Tyrosine**	180.06485	M-H	180.0666	HMDB0000158	C9H11NO3	Carboxylic acids and derivatives	Amino acids, peptides, and analogues	-0.0297246	1.50835	1.841172735
**Citrulline**	176.10354	M+H	176.103	HMDB0000904	C6H13N3O3	Carboxylic acids and derivatives	Amino acids, peptides, and analogues	-0.0902123	1.93838	2.035556757
**Valine**	118.08697	M+H	118.0863	HMDB0000883	C5H11NO2	Carboxylic acids and derivatives	Amino acids, peptides, and analogues	-0.0246663	2.12684	2.499130892
**Leucine**	132.10254	M+H	132.1019	HMDB0000687	C6H13NO2	Carboxylic acids and derivatives	Amino acids, peptides, and analogues	-0.0316081	2.89705	1.633339163
**Betaine**	118.08693	M+H	118.0868	HMDB0000043	C5H11NO2	Carboxylic acids and derivatives	Amino acids, peptides, and analogues	-0.138779	3.00433	2.171733215

**L-Norleucine**	132.10252	M+H	132.1019	HMDB0001645	C6H13NO2	Carboxylic acids and derivatives	Amino acids, peptides, and analogues	-0.121679	3.20619	2.477752762
**L-Isoleucine**	132.10254	M+H	132.1019	HMDB0000172	C6H13NO2	Carboxylic acids and derivatives	Amino acids, peptides, and analogues	-0.0484952	3.268	4.670748949
**L-Phenylalanine**	166.08683	M+H	166.0863	HMDB0000159	C9H11NO2	Carboxylic acids and derivatives	Amino acids, peptides, and analogues	-0.0990585	3.75791	1.511466572
**Hypogeic acid**	253.216	M-H	253.2173	HMDB0002186	C16H30O2	Fatty Acyls	Fatty acids and conjugates	-0.017491	1.02884	1.640680819
**9,10-DHOME**	313.23756	M-H	313.2384	HMDB0004704	C18H34O4	Fatty Acyls	Fatty acids and conjugates	-0.0532029	1.085	9.582987285
**Palmitoleic acid**	272.25986	M+NH4	272.2584	HMDB0003229	C16H30O2	Fatty Acyls	Fatty acids and conjugates	-0.0322463	1.09118	4.860667687
**Palmitic Acid**	255.23176	M-H	255.233	HMDB0000220	C16H32O2	Fatty Acyls	Fatty acids and conjugates	-0.140272	4.33052	1.994949757
**Oleic Acid**	281.24737	M-H	281.2486	HMDB0000207	C18H34O2	Fatty Acyls	Fatty acids and conjugates	-0.510948	9.29229	3.73618034
**O-Arachidonoyl ethanolamine**	348.28839	M+H	348.2897	HMDB0013655	C22H37NO2	Fatty Acyls	Fatty acid esters	-0.0412803	1.1085	71.45129225
**9-Hpode**	311.22199	M-H	311.2228	HMDB0242602	C18H32O4	Fatty Acyls	Lineolic acids and derivatives	-0.0858549	1.62374	5.058564105
**Linoleic acid**	279.23178	M-H	279.233	HMDB0000673	C18H32O2	Fatty Acyls	Lineolic acids and derivatives	-0.0721903	2.2602	1.853730284

**9-Oxo-ODE**	317.20952	M+Na	317.2087	HMDB0004669	C18H30O3	Fatty Acyls	Lineolic acids and derivatives	-0.14141	2.89206	4.142822736
**α-Eleostearic acid/Punicic acid**	279.23253	M+H	279.2319	HMDB0030963	C18H30O2	Fatty Acyls	Lineolic acids and derivatives	-0.181228	3.34584	6.634773927
**13-HOTE**	293.21113	M-H	293.2122	HMDB0010203	C18H30O3	Fatty Acyls	Lineolic acids and derivatives	-0.234664	4.54629	10.92244004
**13-HODE**	295.22649	M-H	295.2279	HMDB0004667	C18H32O3	Fatty Acyls	Lineolic acids and derivatives	-0.302645	5.64255	9.813644291
**Apigenin**	271.06068	M+H	271.0601	HMDB0002124	C15H10O5	Flavonoids	Flavones	-0.0567192	1.67306	4.763999537
**5,7-Dihydroxyflavone**	255.0656	M+H	255.0652	HMDB0036619	C15H10O4	Flavonoids	Flavones	-0.0901933	1.86161	5.937257787
**Monoelaidin**	379.28268	M+Na	379.2819	HMDB0254854	C21H40O4	Glycerolipids	Monoradylglycerols	-0.06785	2.46256	1.752579037
**trans-3-Indoleacrylic acid**	188.07149	M+H	188.0706	HMDB0000734	C11H9NO2	Indoles and derivatives	Indoles	-0.0813946	1.69849	2.696539495
**DL-Tryptophan**	188.07136	/	/	HMDB0013609	C11H12N2O2	Indoles and derivatives	Indolyl carboxylic acids and derivatives	-0.0912294	1.94363	3.046618583
**1H-Indole-4-carboxaldehyde**	146.06061	M+H	146.06	HMDB0341228	C9H7NO	Indoles and derivatives	Indoles	-0.021973	1.12424	7.133127333
**Daidzein**	253.04947	M-H	253.0506	HMDB0003312	C15H10O4	Isoflav-2-enes	Isoflavones	-0.112613	2.34255	6.862896193

**Genistein**	269.04464	M-H	269.0455	HMDB0003217	C15H10O5	Isoflavonoids	Isoflav-2-enes	-0.222719	4.39589	6.893296771
**Trihexyphenidyl**	302.24634	M+H	302.2478	HMDB0014520	C20H31NO	Organonitrogen compounds	Amines	-0.0528789	1.06106	3.023143865
**Oleoyl ethanolamide**	348.28829	/	/	HMDB0002088	C20H39NO2	Organonitrogen compounds	Amines	-0.0778819	2.25478	3.113164114
**Linoleoyl Ethanolamide**	346.27254	M+Na	346.2716	HMDB0012252	C20H37NO2	Organonitrogen compounds	Amines	-0.108528	2.35153	2.927503727
**Choline**	104.10773	M+H	104.1075	HMDB0000097	C5H14NO	Organonitrogen compounds	Quaternary ammonium salts	-0.154172	2.99125	3.690060073
**D-Glucose 6-phosphate**	259.02125	M-H	259.0224	HMDB0001401	C6H13O9P	Organooxygen compounds	Carbohydrates and carbohydrate conjugates	-0.0479712	1.17746	2.309046072
**Epitestosterone**	289.21457	M+H	289.2162	HMDB0000628	C19H28O2	Steroids and steroid derivatives	Androstane steroids	-0.0529154	1.00417	9.784348052
**Ipratropium**	332.22061	M+H	332.222	HMDB0014476	C20H30NO3	Tropane alkaloids	/	-0.0819184	1.59995	1.719365488

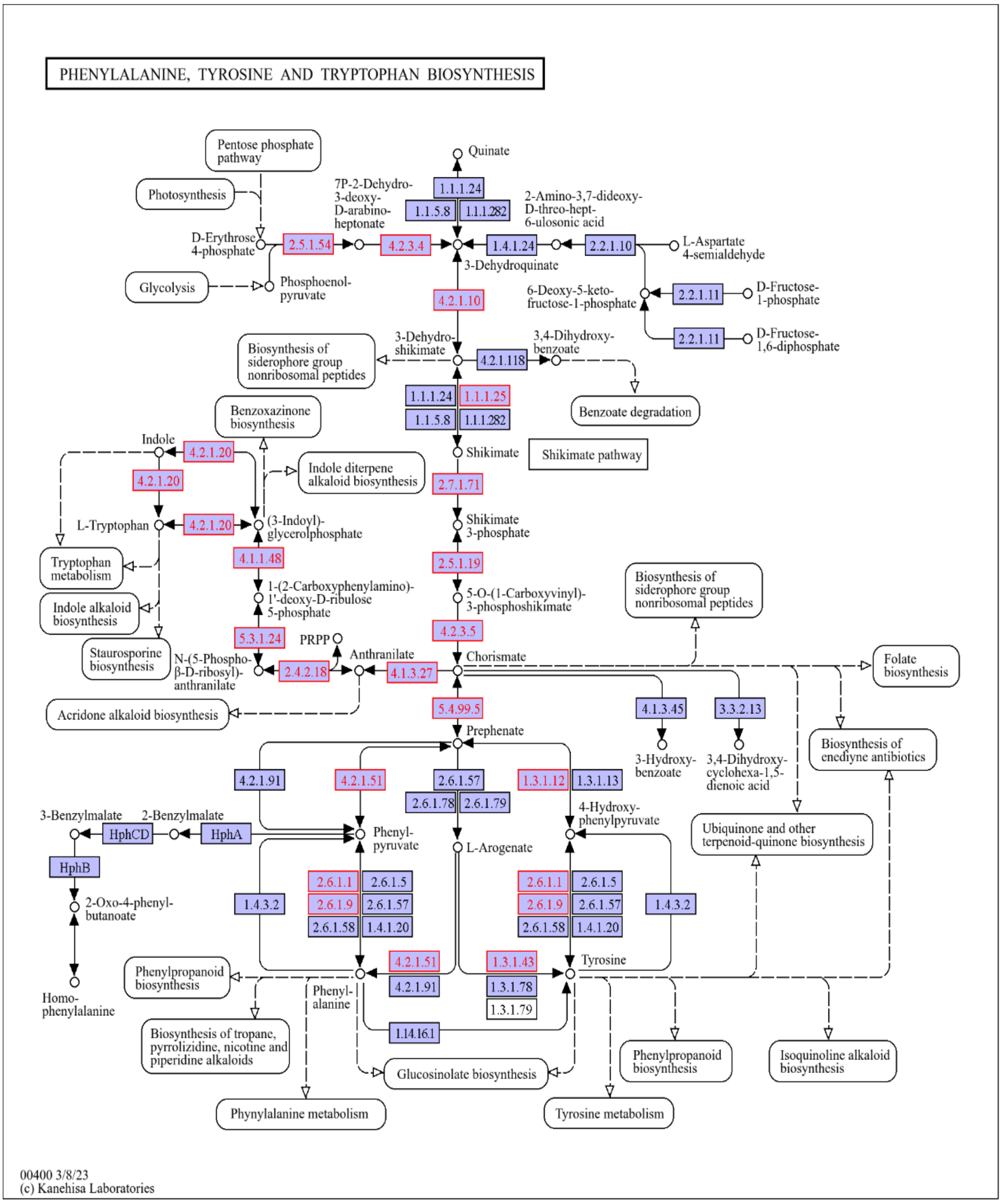
PHENYLALANINE, TYROSINE AND TRYPTOPHAN BIOSYNTHESIS
Pentose phosphate pathway
Photosynthesis
Glycolysis
D-Erythrose 4-phosphate
Phosphoenol-pyruvate
2.5.1.54
7P-2-Dehydro-3-deoxy-D-arabino-heptonate
4.2.3.4
Quinate
1.1.1.24
1.1.5.8
1.1.1.282
2-Amino-3,7-dideoxy-D-threo-hept-6-ulosonic acid
1.4.1.24
2.2.1.10
L-Aspartate 4-semialdehyde
3-Dehydroquinate
4.2.1.10
6-Deoxy-5-keto-fructose-1-phosphate
2.2.1.11
D-Fructose-1-phosphate
2.2.1.11
D-Fructose-1,6-diphosphate
Biosynthesis of siderophore group nonribosomal peptides
3-Dehydro-shikimate
4.2.1.118
3,4-Dihydroxy-benzoate
Benzoate degradation
1.1.1.24
1.1.1.25
1.1.5.8
1.1.1.282
Benzoxazinone biosynthesis
Shikimate
Shikimate pathway
Indole
4.2.1.20
4.2.1.20
Indole diterpene alkaloid biosynthesis
2.7.1.71
L-Tryptophan
4.2.1.20
(3-Indoyl)-glycerolphosphate
Shikimate 3-phosphate
Tryptophan metabolism
4.1.1.48
2.5.1.19
Indole alkaloid biosynthesis
1-(2-Carboxyphenylamino)-1'-deoxy-D-ribulose 5-phosphate
5-O-(1-Carboxyvinyl)-3-phosphoshikimate
Biosynthesis of siderophore group nonribosomal peptides
5.3.1.24
PRPP
4.2.3.5
Staurosporine biosynthesis
N-(5-Phospho-β-D-ribosyl)-anthranilate
2.4.2.18
Anthranilate
4.1.3.27
Chorismate
Folate biosynthesis
Acridone alkaloid biosynthesis
5.4.99.5
4.1.3.45
3.3.2.13
Prephenate
3-Hydroxy-benzoate
3,4-Dihydroxy-cyclohexa-1,5-dienoic acid
Biosynthesis of enediyne antibiotics
4.2.1.91
4.2.1.51
2.6.1.57
1.3.1.12
1.3.1.13
2.6.1.78
2.6.1.79
4-Hydroxy-phenylpyruvate
3-Benzylmalate
2-Benzylmalate
HphCD
HphA
Phenyl-pyruvate
L-Arogenate
Ubiquinone and other terpenoid-quinone biosynthesis
HphB
2.6.1.1
2.6.1.5
2.6.1.1
2.6.1.5
2-Oxo-4-phenyl-butanoate
1.4.3.2
2.6.1.9
2.6.1.57
2.6.1.9
2.6.1.57
1.4.3.2
2.6.1.58
1.4.1.20
2.6.1.58
1.4.1.20
4.2.1.51
1.3.1.43
Tyrosine
Phenylpropanoid biosynthesis
4.2.1.91
1.3.1.78
Homo-phenylalanine
Phenyl-alanine
1.3.1.79
Biosynthesis of tropane, pyrrolizidine, nicotine and piperidine alkaloids
1.14.16.1
Phenylpropanoid biosynthesis
Isoquinoline alkaloid biosynthesis
Glucosinolate biosynthesis
Phynylalanine metabolism
Tyrosine metabolism
00400 3/8/23
(c) Kanehisa Laboratories

A

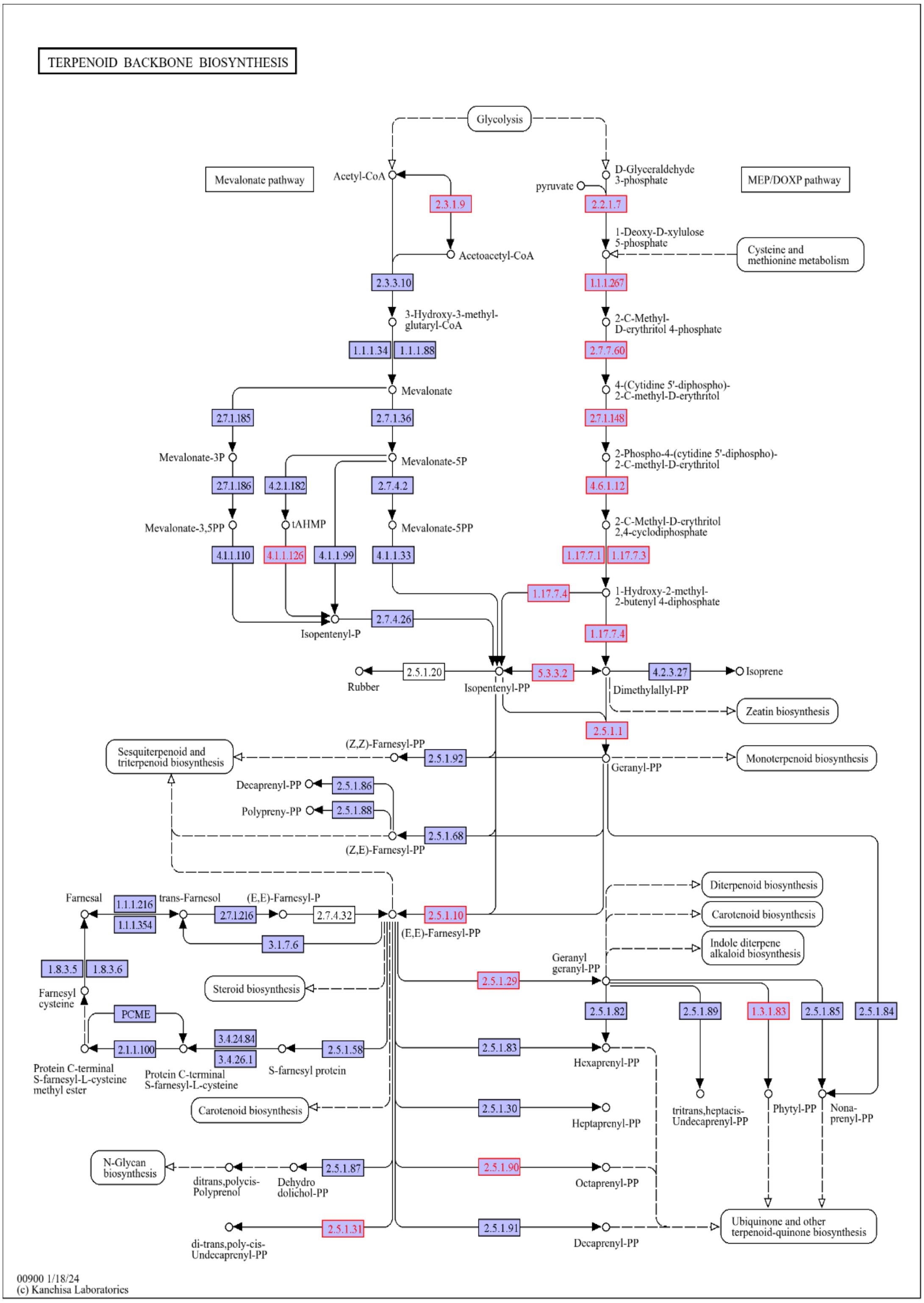
TERPENOID BACKBONE BIOSYNTHESIS
Glycolysis
Mevalonate pathway
Acetyl-CoA
2.3.1.9
Acetoacetyl-CoA
2.3.3.10
3-Hydroxy-3-methyl-
glutaryl-CoA
1.1.1.34
1.1.1.88
Mevalonate
2.7.1.185
2.7.1.36
Mevalonate-3P
2.7.1.186
4.2.1.182
Mevalonate-5P
2.7.4.2
Mevalonate-3,5PP
tAHMP
Mevalonate-5PP
4.1.1.110
4.1.1.126
4.1.1.99
4.1.1.33
2.7.4.26
Isopentenyl-P
pyruvate
D-Glyceraldehyde
3-phosphate
MEP/DOXP pathway
2.2.1.7
1-Deoxy-D-xylulose
5-phosphate
Cysteine and
methionine metabolism
1.1.1.267
2-C-Methyl-
D-erythritol 4-phosphate
2.7.7.60
4-(Cytidine 5'-diphospho)-
2-C-methyl-D-erythritol
2.7.1.148
2-Phospho-4-(cytidine 5'-diphospho)-
2-C-methyl-D-erythritol
4.6.1.12
2-C-Methyl-D-erythritol
2,4-cyclodiphosphate
1.17.7.1
1.17.7.3
1.17.7.4
1-Hydroxy-2-methyl-
2-butenyl 4-diphosphate
1.17.7.4
Rubber
2.5.1.20
Isopentenyl-PP
5.3.3.2
Dimethylallyl-PP
4.2.3.27
Isoprene
Zeatin biosynthesis
2.5.1.1
Sesquiterpenoid and
triterpenoid biosynthesis
(Z,Z)-Farnesyl-PP
2.5.1.92
Geranyl-PP
Monoterpenoid biosynthesis
Decaprenyl-PP
2.5.1.86
Polypreny-PP
2.5.1.88
2.5.1.68
(Z,E)-Farnesyl-PP
Farnesal
1.1.1.216
1.1.1.354
trans-Farnesol
(E,E)-Farnesyl-P
2.7.1.216
2.7.4.32
2.5.1.10
(E,E)-Farnesyl-PP
3.1.7.6
Diterpenoid biosynthesis
Carotenoid biosynthesis
Indole diterpene
alkaloid biosynthesis
1.8.3.5
1.8.3.6
Farnesyl
cysteine
Steroid biosynthesis
2.5.1.29
Geranyl
geranyl-PP
PCME
3.4.24.84
3.4.26.1
2.1.1.100
2.5.1.58
Protein C-terminal
S-farnesyl-L-cysteine
methyl ester
Protein C-terminal
S-farnesyl-L-cysteine
S-farnesyl protein
2.5.1.82
2.5.1.89
1.3.1.83
2.5.1.85
2.5.1.84
2.5.1.83
Hexaprenyl-PP
Carotenoid biosynthesis
2.5.1.30
Heptaprenyl-PP
tritrans,heptacis-
Undecaprenyl-PP
Phytyl-PP
Nona-
prenyl-PP
N-Glycan
biosynthesis
ditrans,polycis-
Polyprenol
Dehydro
dolichol-PP
2.5.1.87
2.5.1.90
Octaprenyl-PP
Ubiquinone and other
terpenoid-quinone biosynthesis
di-trans,poly-cis-
Undecaprenyl-PP
2.5.1.31
2.5.1.91
Decaprenyl-PP
00900 1/18/24
(c) Kanehisa Laboratories

B

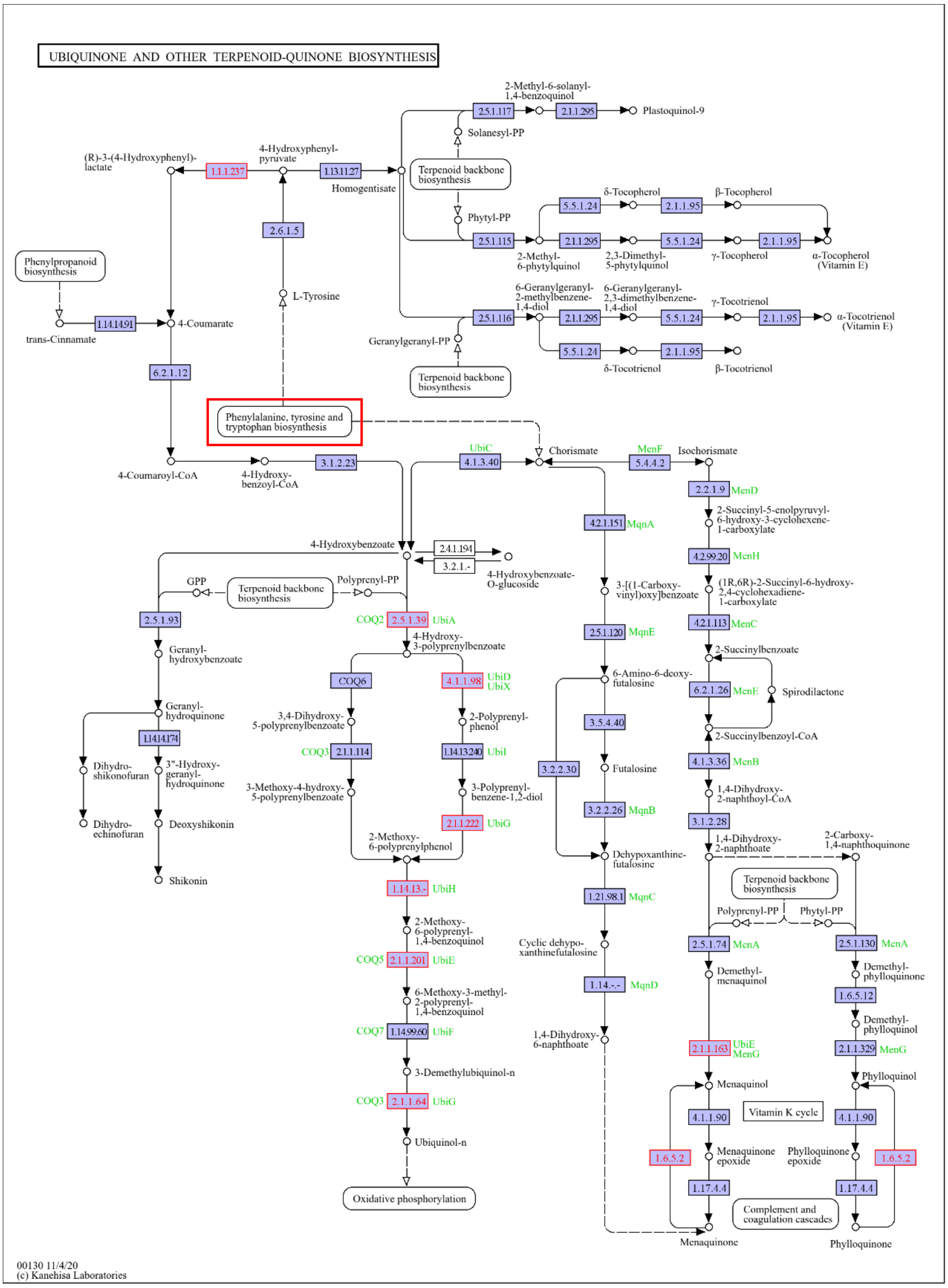

C

Figure S3. The information of gene annotation pathways in KEGG database related to Coenzyme Q10 biosynthesis, **A** Gene annotation in phenylalanine, tyrosine and tryptophan biosynthesis pathway, **B** Gene annotation in terpenoid backbone biosynthesis pathway, **C** Gene annotation in the ubiquinone and other terpenoid-quinone biosynthesis pathway (phenylalanine, tyrosine, and tryptophan biosynthesis was marked with a red box).

Table S5. The results of pathway analysis.

Compounds	Total	Expected	Hits	Raw p	-LOG10(p)	Holm adjust	FDR	Impact
**Valine, leucine and isoleucine biosynthesis**	8	0.07619	3	3.81E-05	4.4191	0.0030476	0.0030476	0
**Phenylalanine, tyrosine and tryptophan biosynthesis**	4	0.038095	2	0.00050267	3.2987	0.039711	0.020107	1
**Phenylalanine metabolism**	8	0.07619	2	0.0022946	2.6393	0.17898	0.061189	0.35714
**Biosynthesis of unsaturated fatty acids**	36	0.34286	3	0.0041344	2.3836	0.31835	0.082687	0
**Valine, leucine and isoleucine degradation**	40	0.38095	3	0.0055906	2.2525	0.42488	0.089449	0
**Neomycin, kanamycin and gentamicin biosynthesis**	2	0.019048	1	0.018963	1.7221	1	0.25284	0
**Glycine, serine and threonine metabolism**	33	0.31429	2	0.037709	1.4236	1	0.43096	0.05149
**Linoleic acid metabolism**	5	0.047619	1	0.046779	1.3299	1	0.46779	1
**Arginine biosynthesis**	14	0.13333	1	0.12587	0.90006	1	1	0.22843
**Ubiquinone and other terpenoid-quinone biosynthesis**	18	0.17143	1	0.15902	0.79854	1	1	0
**Starch and sucrose metabolism**	18	0.17143	1	0.15902	0.79854	1	1	0.13851
**Pantothenate and CoA biosynthesis**	20	0.19048	1	0.17515	0.75658	1	1	0
**Glutathione metabolism**	28	0.26667	1	0.23683	0.62556	1	1	0.02698
**Lysine degradation**	30	0.28571	1	0.25157	0.59935	1	1	0
**Inositol phosphate metabolism**	30	0.28571	1	0.25157	0.59935	1	1	0
**Glycerophospholipid metabolism**	36	0.34286	1	0.29419	0.53138	1	1	0.02582
**Fatty acid elongation**	39	0.37143	1	0.31464	0.50219	1	1	0

**Fatty acid degradation**	39	0.37143	1	0.31464	0.50219	1	1	0
**Tyrosine metabolism**	42	0.4	1	0.33453	0.47556	1	1	0.13972
**Fatty acid biosynthesis**	47	0.44762	1	0.3665	0.43592	1	1	0.01473
**Steroid hormone biosynthesis**	87	0.82857	1	0.57525	0.24014	1	1	0.03757

Table S6. The detailed gene annotation information of strain YS02.

See the excel in the zip document.
